# When Steroids Backfire: Tinea Incognito Following Treatment for Presumed Psoriasis

**DOI:** 10.7759/cureus.116671

**Published:** 2026-09-21

**Authors:** Nicole Skalka, Nathaniel Marroquin, Jay Nguyen, Panagiotis Mitropoulos, Patrick Dominguez

**Affiliations:** 1 Dermatology, Rocky Vista University College of Osteopathic Medicine, Ivins, USA; 2 Dermatology, Kansas City University-Graduate Medical Education Consortium, Advanced Dermatology and Cosmetic Surgery-Orlando Program, Maitland, USA

**Keywords:** case report, dermatophyte, potassium hydroxide, psoriasis, tinea incognito

## Abstract

Tinea incognito is an altered clinical presentation of a dermatophyte infection that develops following treatment with topical or systemic corticosteroids or other immunosuppressive agents. Suppression of inflammation temporarily improves the appearance of the eruption while masking the characteristic annular, scaly appearance of the infection, making diagnosis more challenging. If a fungal infection is suspected, a potassium hydroxide (KOH) preparation should be performed to evaluate for an underlying fungal etiology. A 43-year-old male presented to dermatology after treatment with clobetasol propionate 0.05% cream and roflumilast 0.3% cream for presumed psoriasis. His eruption initially improved but subsequently recurred and progressively worsened over three months. KOH preparation revealed fungal hyphae, leading to the diagnosis of tinea incognito. This case highlights the importance of maintaining a high index of suspicion for fungal infection in patients presenting with a scaly eruption and underscores the value of diagnostic tools, such as KOH preparation, prior to initiating corticosteroids or other immunosuppressive agents.

## Introduction

Dermatophyte infections, commonly known as "ringworm," are superficial fungal infections named for their characteristic ring-shaped (annular) appearance rather than an underlying parasitic worm [1]. Although dermatophyte infections often present in the pediatric population, they can occur among patients of all ages [2]. Tinea corporis, a dermatophyte infection of the body, typically presents as erythematous annular plaques with overlying scale and central clearing [3]. Common dermatophyte infections include tinea cruris (groin), tinea pedis (feet), tinea capitis (scalp), tinea manuum (palm), and onychomycosis (nails) [2,3]. When dermatophyte infections are misdiagnosed and treated with systemic or topical corticosteroids, calcineurin inhibitors, or other immunosuppressive agents, their clinical appearance may become altered, resulting in tinea incognito [2,3]. The discovery of tinea incognito was first described by two dermatologists, Dr. Ive and Dr. Marks, in 1968 [4]. They recognized the ability of topical corticosteroids or other immunosuppressive agents to mask fungal infections, and the term “tinea incognita” was developed [4].

Tinea incognito can be misdiagnosed because its altered clinical morphology may mimic inflammatory dermatoses [1]. Delayed recognition may allow the infection to persist or progress while inappropriate therapy is continued [2]. A simple and affordable method to diagnose cutaneous fungal infections is direct microscopy with potassium hydroxide (KOH) preparation. The presence of hyphae is diagnostic of fungal infection [2]. This case highlights tinea incognito as a diagnostic challenge and reinforces the importance of simple in-office testing, such as KOH preparation, to establish the correct diagnosis. These findings underscore the importance of confirming the diagnosis before initiating empiric or combination antifungal-corticosteroid therapy to ensure targeted treatment and avoid masking an underlying dermatophyte infection.

## Case presentation

A 43-year-old male presented to dermatology with a broad, well-demarcated erythematous, scaly plaque involving the upper back, posterior neck, and occipital region (Figure 1). He had previously been evaluated by his primary care provider for a similar eruption and was diagnosed with psoriasis, for which he was treated with clobetasol propionate 0.05% cream and roflumilast 0.3% cream. The patient reported that the eruption initially subsided with these topical therapies. However, it recurred and progressively worsened over the subsequent three months.

**Figure 1:**
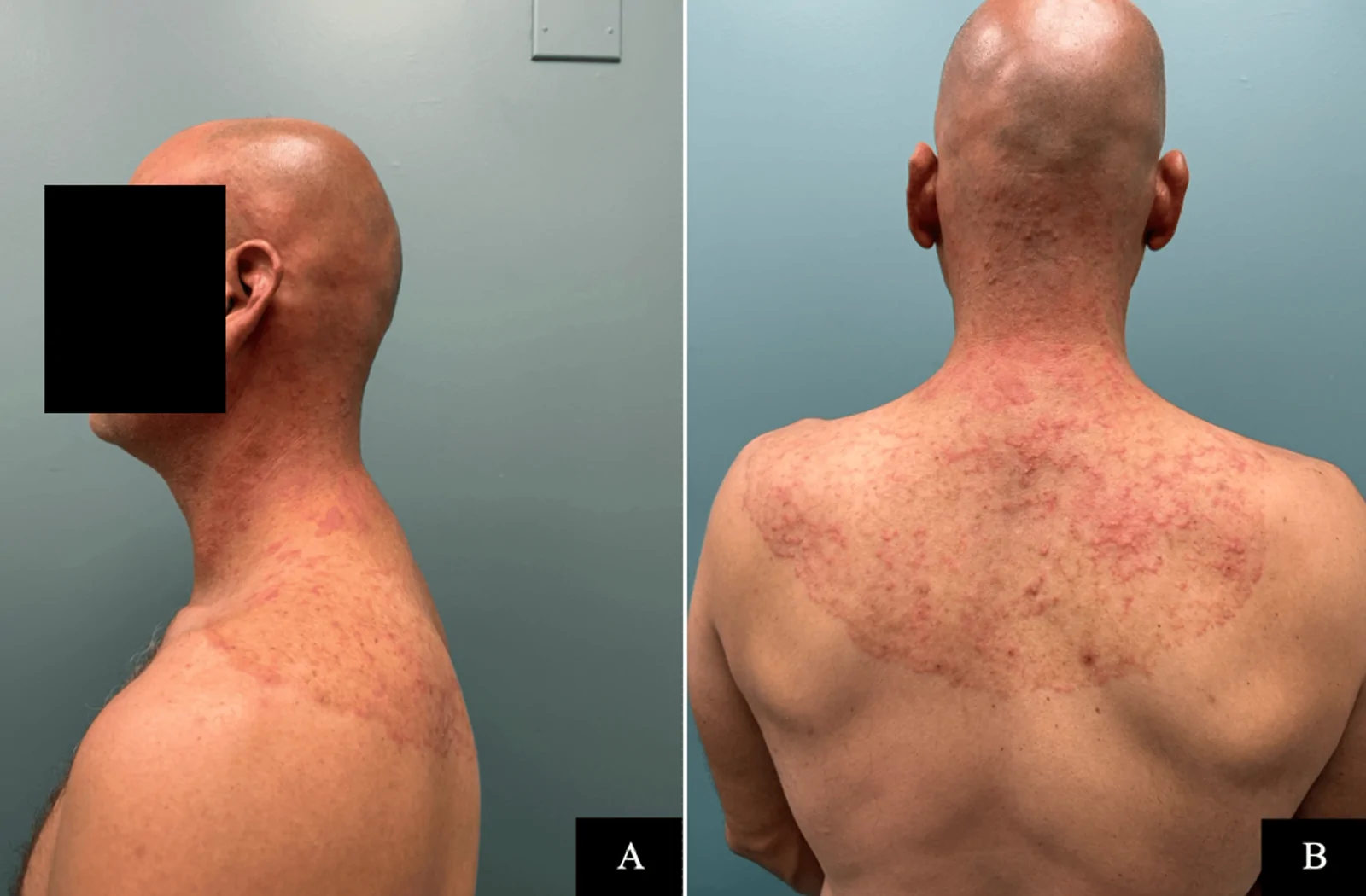
Clinical photographs demonstrating a broad, well-demarcated erythematous, scaly plaque involving the posterior neck and upper back at initial dermatologic presentation. (A) Lateral view and (B) posterior view.

Further evaluation included a KOH preparation, which demonstrated fungal hyphae (Figure 2). Based on these in-office lab findings and clinical history of treatment with topical corticosteroids, a diagnosis of tinea incognito was made. The patient was instructed to discontinue clobetasol propionate 0.05% cream and roflumilast 0.3% cream and was treated with econazole nitrate cream 1% twice daily and terbinafine HCL 250 mg daily by mouth for three weeks. The patient was subsequently lost to follow-up. 

**Figure 2:**
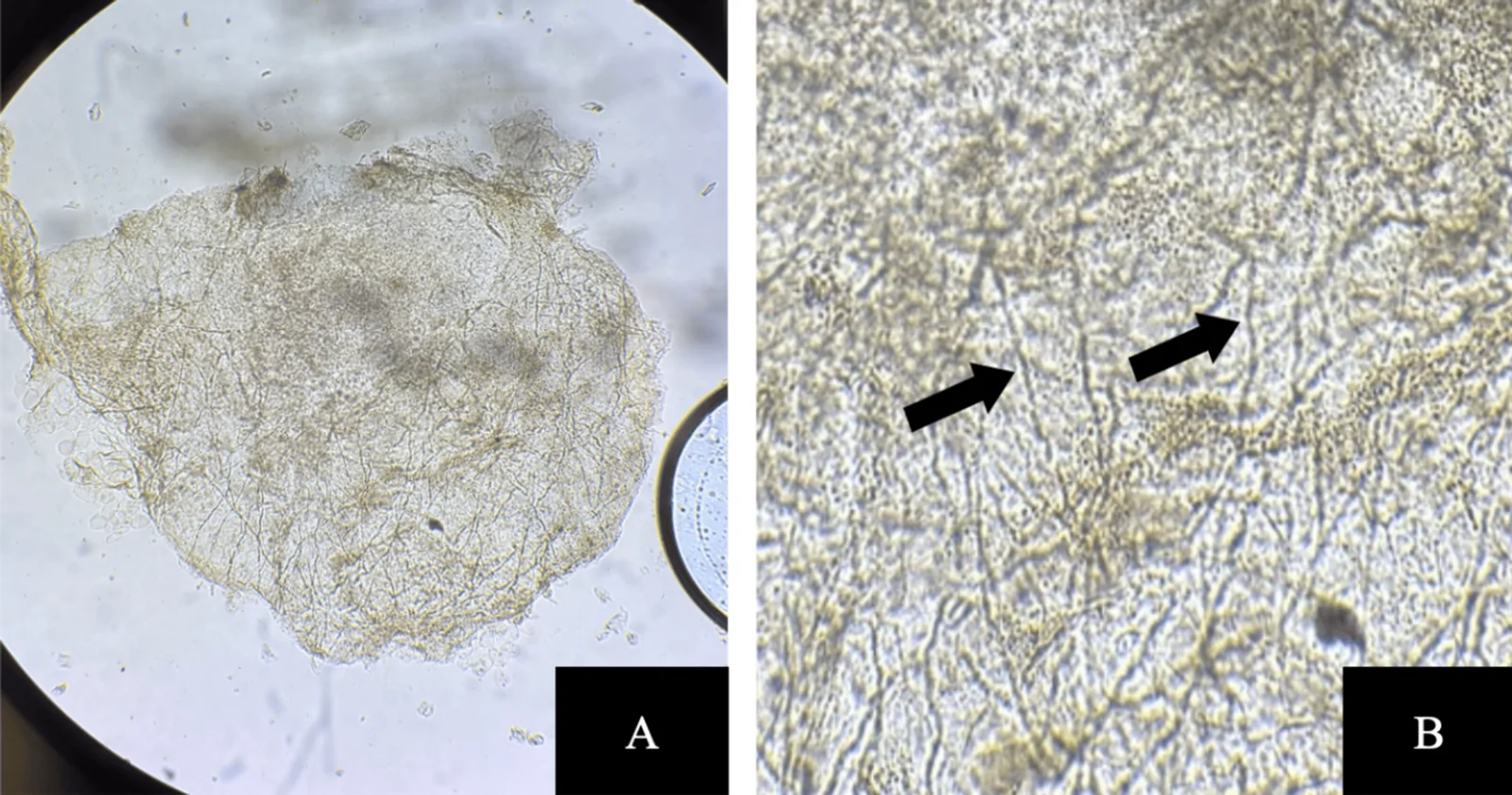
Potassium hydroxide (KOH) preparation demonstrating fungal hyphae, characteristic of dermatophyte infection. (A) Low-power view at 2.5× magnification. (B) Higher-power view at 40× magnification demonstrating fungal hyphae (black arrows).

## Discussion

When a potential cutaneous fungal infection is suspected, consider using a simple point-of-care diagnostic tool such as a KOH preparation. The KOH preparation technique has minimal requirements: a KOH solution, a microscope, a glass slide, a cover slip, and a blade to scrape and collect a sample. This technique is performed by scraping scale from the plaque onto a glass slide, followed by the application of a 20% KOH solution to the specimen to break down keratin and cellular debris. The preparation may then be allowed to sit briefly or be gently warmed to facilitate keratin dissolution before microscopic examination. Additional reagents may include chlorazol black, acridine orange, or trypan blue. This process allows for clear visualization of fungal elements, including hyphae and spores, under microscopy [5]. A key advantage of KOH preparation is that it is significantly faster and more cost-effective, providing diagnostic results within minutes rather than weeks [6]. In clinical settings where KOH microscopy is not readily available, fungal culture or referral for additional diagnostic evaluation may be considered when the diagnosis remains uncertain.

The differential diagnosis for tinea incognito includes psoriasis, eczema, cutaneous lupus, impetigo, and seborrheic dermatitis [1]. In fact, it is most commonly recognized in patients initially treated for inflammatory dermatoses such as psoriasis with topical therapies [1]. This was also observed in our patient, whose eruption was initially diagnosed and treated as psoriasis before KOH examination demonstrated fungal hyphae.

The development of tinea incognito begins when a dermatophyte infection is initially treated with topical or systemic corticosteroids or other immunosuppressive agents before the fungal etiology is recognized. In this case, the patient received both clobetasol propionate and roflumilast concurrently. Topical corticosteroid exposure is a well-established precipitating factor in tinea incognito. Because the therapies were used concomitantly, the independent contribution of roflumilast to the altered clinical presentation cannot be determined. Topical corticosteroids may initially improve the appearance of the eruption by suppressing inflammation. However, they may also mask the characteristic annular, scaly appearance of dermatophyte infection. This masking effect allows the underlying fungal infection to persist and progress, ultimately resulting in the altered clinical presentation known as tinea incognito [3]. 

In this case, the initial improvement with anti-inflammatory therapy may have provided false reassurance and contributed to delayed recognition of the underlying dermatophyte infection. Earlier consideration of a fungal etiology and use of KOH preparation may have helped establish the diagnosis before additional anti-inflammatory therapy was continued. In primary care settings where KOH microscopy is available, point-of-care testing may help clarify the diagnosis of persistent or atypical scaly eruptions.

Reducing misdiagnosis requires maintaining clinical suspicion for dermatophyte infection and using appropriate point-of-care testing, such as KOH preparation, when indicated. In addition to KOH preparation, diagnostic pearls may help differentiate commonly confused skin conditions. Pruritus may occur with tinea infection, but it is also common in psoriasis and should not be used in isolation to distinguish between these conditions. Psoriasis may also present with nail pitting and plaques with a silvery scale on the extensor surfaces, scalp, trunk, intergluteal cleft, and nails. A helpful clue for atopic dermatitis is a personal or family history of atopy, including atopic dermatitis, asthma, or allergic rhinitis. Tinea infections are also more common in patients with a history of contact sports or household pets. 

Broader use of this diagnostic tool may help reduce inappropriate treatment and diagnostic delay. If tinea incognito is confirmed, corticosteroid or other immunosuppressive therapy contributing to the altered presentation should be discontinued when clinically appropriate [2]. The most common causative organisms of tinea corporis are *Trichophyton rubrum*, followed by *Trichophyton mentagrophytes*. Systemic antifungal therapy may be considered for extensive or refractory dermatophyte infection, involvement of multiple sites, or when topical therapy alone is unlikely to be sufficient [2]. Antifungal agents are broadly classified as fungistatic, which inhibit fungal growth, or fungicidal, which directly kill fungal cells. These medications target the ergosterol synthesis pathway at different enzymatic steps. First-line treatment for localized dermatophyte infections includes topical antifungals such as ketoconazole, whereas systemic options include oral agents such as fluconazole or terbinafine. Because the patient was lost to follow-up, clinical and mycologic response to antifungal therapy could not be assessed. In addition, fungal culture and organism identification were not obtained.

## Conclusions

This case highlights the importance of maintaining a high index of suspicion for fungal infection in patients presenting with a scaly eruption in the outpatient setting. Early use of simple diagnostic tools, such as KOH preparation, may help reduce delayed diagnosis and inappropriate therapy. Clinicians should consider KOH preparation before initiating immunosuppressive therapies when an infectious eruption remains in the differential diagnosis.
